# Proceedings from the Fourth International Symposium on σ-2 Receptors: Role in Health and Disease

**DOI:** 10.1523/ENEURO.0317-20.2020

**Published:** 2020-11-04

**Authors:** Nicholas J. Izzo, Martí Colom-Cadena, Aladdin A. Riad, Jinbin Xu, Meharvan Singh, Carmen Abate, Michael A. Cahill, Tara L. Spires-Jones, Wayne D. Bowen, Robert H. Mach, Susan M. Catalano

**Affiliations:** 1 Cognition Therapeutics Inc, Pittsburgh, PA, 15203; 2 UK Dementia Research Institute and The University of Edinburgh, Edinburgh, United Kingdom, EH8 9JZ; 3 The University of Pennsylvania, Department of Radiology, Philadelphia, PA, 19104; 4 Washington University School of Medicine, Department of Radiology, St. Louis, MO, 63110; 5 Loyola University Chicago Stritch School of Medicine, Department of Cellular and Molecular Physiology, Maywood, IL, 60153; 6 Dipartimento di Farmacia-Scienze del Farmaco, Università degli Studi di Bari, I-70125 Bari, Italy; 7 School of Biomedical Sciences, Charles Sturt University, Wagga Wagga, New South Wales, 2650 Australia; 8 Department of Cancer Biology and Therapeutics, John Curtin School of Medical Research, Australian National University, Canberra, Australian Capital Territory, 2601 Australia; 9 Department of Molecular Pharmacology, Physiology and Biotechnology, Brown University, Providence, RI 02912

**Keywords:** amyloid β, neurodegeneration, PGRMC1, SfN symposium, σ-2 receptor, TMEM97

## Abstract

The σ-2 receptor (S2R) complex has been implicated in CNS disorders ranging from anxiety and depression to neurodegenerative disorders such as Alzheimer’s disease (AD). The proteins comprising the S2R complex impact processes including autophagy, cholesterol synthesis, progesterone signaling, lipid membrane-bound protein trafficking, and receptor stabilization at the cell surface. While there has been much progress in understanding the role of S2R in cellular processes and its potential therapeutic value, a great deal remains unknown. The International Symposium on Sigma-2 Receptors is held in conjunction with the annual Society for Neuroscience (SfN) conference to promote collaboration and advance the field of S2R research. This review summarizes updates presented at the Fourth International Symposium on Sigma-2 Receptors: Role in Health and Disease, a Satellite Symposium held at the 2019 SfN conference. Interdisciplinary members of the S2R research community presented both previously published and preliminary results from ongoing studies of the role of S2R in cellular metabolism, the anatomic and cellular expression patterns of S2R, the relationship between S2R and amyloid β (Aβ) in AD, the role of S2R complex protein PGRMC1 in health and disease, and the efforts to design new S2R ligands for the purposes of research and drug development. The proceedings from this symposium are reported here as an update on the field of S2R research, as well as to highlight the value of the symposia that occur yearly in conjunction with the SfN conference.

## Introduction

The σ receptors were first identified in the 1970s and were initially believed to be opioid receptors because of the CNS effects of their ligand SKF10047; they were recognized as distinct when opiate antagonists were found not to block their activity (Martin et al., 1976). The σ-2 receptor (S2R) was recognized apart from σ-1 (S1R) in the early 1990s (Hellewell and Bowen, 1990). Since then, σ receptors have continued to be of interest in the field of neuroscience because of effects in schizophrenia, depression and anxiety, pain, and neuroprotection (for review of σ receptor history and nomenclature, see Zeng and Mach, 2017). Despite the broad potential relevance of S2Rs to the treatment of these neurologic conditions, no selective S2R compound has yet achieved clinical success. The absence of an identified protein corresponding to S2R hampered progress toward understanding its role in the brain for many years. In 2011, the S2R complex was found to contain the PGRMC1 protein complex (Xu et al., 2011). This discovery expanded the field of S2R research to include the effects of PGRMC1, among which are impacts on oxic/glycolytic and sterol metabolism, membrane trafficking, growth factor release, axon guidance in neurodevelopment, and neuroprotection (Su et al., 2012; Kabe et al., 2016; Nicholson et al., 2016; Cahill, 2017; Cahill and Medlock, 2017).

Studies of this complex accelerated with the identification of TMEM97 as a gene coding for S2R activity in tumor cell lines in 2017 (Alon et al., 2017). In the following, we refer to undefined S2R ligand binding activity as S2R and functions known to involve TMEM97 as S2R/TMEM97. S2R/TMEM97 has now been shown to play a role in cellular damage response mechanisms, and its constituent proteins regulate processes, including autophagy, cholesterol synthesis and progesterone signaling, lipid membrane-bound protein trafficking, and receptor stabilization at the cell surface (Cahill, 2007; Ahmed et al., 2012; Su et al., 2012; Cahill et al., 2016; Nguyen et al., 2018; Riad et al., 2018; Oyer et al., 2019). Additional binding partners for S2R/TMEM97 have been determined, notably the low-density lipoprotein receptor (LDLR), an interaction that has implications for lipid homeostasis and Alzheimer’s disease (AD; Riad et al., 2018). Furthermore, S2R/TMEM97 has most recently been identified as a binding partner for SARS-CoV-2, the novel coronavirus, as it interacts with the viral protein orf9c, while the S1R interacts with Nsp6 (Gordon et al., 2020).

Despite the progress that has been made in the understanding of the importance of S2R and its receptor complex proteins in cellular processes and its potential value as a therapeutic target in a variety of diseases, a great deal remains unknown. For instance, some S2R ligand activity is independent of TMEM97 (Zeng et al., 2019). Notably, there are few selective S2R small molecule agonist and antagonists that are widely available for research purposes, and the relationship between S2R, TMEM97, PGRMC1, and other binding partners is still coming into focus.

In an effort to promote collaboration and collective understanding of this receptor complex, the International Symposium on Sigma-2 Receptors is held in conjunction with the annual Society for Neuroscience (SfN) conference. Here, we summarize both published and preliminary updates to the field of S2R research that were presented at the Fourth International Symposium on Sigma-2 Receptors: Role in Health and Disease, a SfN Satellite Symposium, held in October 2019. Results presented at the symposium that have not since been published in the peer reviewed literature are not discussed here or are indicated as preliminary.

## Metabolic Effects of S2R Ligands

S2R agonists have traditionally been characterized as ligands that induce programmed cell death in various cell types through a number of mechanisms (Crawford and Bowen, 2002; Zeng et al., 2012). Despite this pharmacological profile, a recent study showed that knock-out of TMEM97 did not affect the cytotoxic potency of some S2R ligands, casting doubt on the role of S2R/TMEM97 in previously established cytotoxic effects, and implying the existence of yet unidentified S2R activities (Zeng et al., 2019). Bowen and colleagues have identified analogs of the canonical S2R antagonist SN79. Some of these are able to induce apoptotic cell death, while others display a novel metabolically stimulative effect (Nicholson et al., 2015, 2016, 2019). This metabolic effect is characterized by increased reductive capacity as indicated by stimulation of MTT [3-[4,5 dimethylthiazol-2-yl]-2,5 diphenyltetrazolium bromide] reduction, increase in cellular ATP (Adenosine triphosphate) level, reduction in basal ROS (Reactive Oxygen Species) level, and stabilization of HIF-1α, as determined in human SK-N-SH neuroblastoma cells (Nicholson et al., 2016). Bowen presented ongoing research at the 2019 SfN meeting and at this S2R satellite symposium that further characterizes this prometabolic effect using additional analogs of SN79 (McVeigh et al., 2019). Preliminary findings suggest that CM764, CM571, and WA504 (S2R Ki = 3.5, 21.7, and 2.5 nm, respectively) induced dose-dependent stimulation of MTT reduction by 45%, 33%, and 75%, respectively, at the highest dose examined (30 μm) after a 24-h treatment. Analogs lacking S2R affinity and structural fragments of the active compounds appear not to have this effect. An examination of the time course suggests that it may take 3–6 h of treatment for this stimulative effect to fully develop. These preliminary findings were consistent with the time course for HIF-1α stabilization shown previously (Nicholson et al., 2016). Like some other S2R ligands (Vilner and Bowen, 2000), all three compounds appear to induce a transient and dose-dependent (10 and 30 μm) increase in cytosolic calcium, an effect that was blocked by thapsigargin pretreatment (150 nm), suggesting that the calcium release is derived from stores within the endoplasmic reticulum (ER). Based on evidence that calcium signaling plays a role in inducing expression of HIF-1α, a global regulator of the glycolytic pathway (Li et al., 2012; Divolis et al., 2016), it is possible that the S2R ligands described here impact glycolysis through ER calcium release, a downstream upregulation of HIF-1α, and a resulting upregulation of glycolytic pathways that may have neuroprotective implications such as protection against oxidative and hypoxic stress. If these preliminary findings are confirmed through further ongoing studies, the results may suggest that S2R plays a role in adaptation of cancer tumor cells to hypoxic environments by upregulating Warburg glycolysis. This effect would be consistent with S2R upregulation in cancer cells. However, whether TMEM97 or another related protein with similar pharmacology mediates these effects is still under investigation.

## Expression and Localization of S2R in the Brain

Appreciating the localization of the σ receptors in the human brain is important to understanding their role in health and disease. To this end, Xu and colleagues have performed quantitative autoradiography on postmortem samples. N-[4-(3,4-dihydro-6,7-dimethoxyisoquinolin-2(1H)-yl)butyl]−2-methoxy-5-methyl-benzamide (RHM-1) has high affinity and selectivity for the S2R (Ki < 10 nm) compared with the S1R (ratio > 300; Mach et al., 2004; Xu et al., 2005). Radiolabeled RHM-1 was therefore used to assess the distribution and expression of S2Rs across human brain samples, and Xu presented preliminary findings from these experiments at the S2R symposium. These findings suggest that both the S1R and S2R are extensively distributed across brain regions, and that S2R may be more highly expressed than the S1R in all brain regions (frontal cortex, precommissural caudate and putamen, postcommissural caudate and putamen, nucleus accumbens, globus pallidus, thalamus, and substantial nigra) except the red nucleus, where expression levels of the two were comparable and lower than in the other regions assessed.

Expression of S2R appears from these experiments to be higher in aged brains, a finding that prompted the question of whether S2R plays a role in disorders such as AD and Parkinson’s disease (PD). The authors therefore are also comparing S1R and S2R expression patterns with those of Tau using the selective Tau radioligand [3H]MK6240 in frontal cortex. Preliminary results from aged AD brains (*N* = 7; six females and one male; aged 74–88 years, Tau tangle rating: 4–6) do not suggest an obvious correlation between Tau density and either S1R or S2R expression, but research is still ongoing.

Although these radioligand studies have thus far not revealed a correlation between Tau expression and S2R activity, the laboratory of Spires-Jones is further assessing the subcellular localization of TMEM97 to determine whether it is present at synapses or in close proximity to amyloid β (Aβ) in human AD brain (Hesse et al., 2019). At the S2R symposium, Colom-Cadena of the Spires-Jones laboratory presented preliminary findings, which have since been published, that synaptic fractions that had been biochemically isolated from human temporal cortex contained TMEM97 (Hesse et al., 2019). Furthermore, the presence of TMEM97 in these fractions appears to be higher in samples isolated from AD patient brain (*n* = 7) compared with those from healthy controls (*n* = 7), a result that is supported by preliminary analyses of temporal cortex synapses with high-resolution array tomography; these suggest that TMEM97 is present at both presynaptic and postsynaptic terminals, and in a larger proportion of synapses in AD (*n* = 9) than in control (*n* = 6) brains.

The authors are furthermore using Förster Resonance Energy Transfer (FRET) to visualize colocalization of TMEM97 and Aβ. Initial findings from these experiments suggest the two are in close enough proximity in synapses to generate a FRET signal. If continued investigation confirms these preliminary findings, TMEM97 may be involved in the mediation of Aβ-induced toxicity in AD.

## The S2R Complex and Aβ

The relationship between S2R/TMEM97 and Aβ has been the subject of many recent studies. S2R/TMEM97 has been shown to form a complex with a number of other proteins, including PGRMC1 and LDLR (Riad et al., 2018). This intact trimeric complex is required for efficient uptake of lipoproteins such as LDL and apolipoprotein E (ApoE). The TMEM97-PGRMC1-LDLR trimeric complex was identified in HeLa cells, in primary rat neurons, and in human brain tissue. The ApoE4 isoform is the greatest risk factor associated with developing AD, and ApoE is known to influence the uptake and accumulation of Aβ, a process that eventually leads to synaptic dysfunction and neurodegeneration in AD. Because of this, the laboratory of Mach seeks to determine whether the S2R/TMEM97 complex is necessary for internalization of Aβ and whether disruption of the complex inhibits Aβ uptake.

To this end, Riad of the Mach laboratory presented results, since published, from CRISPR/Cas9 knock-out of TMEM97 or/and PGRMC1 in HeLa cells, as well as the use of small molecule inhibitors of TMEM97 and PGRMC1 in primary rat cortical neurons. Uptake of Aβ42 (monomeric or oligomeric) in the presence or absence of the main ApoE isoforms (ApoE2, ApoE3, and ApoE4) was assessed using ELISA and confocal microscopy. Uptake of Aβ42, ApoE, and the Aβ42/ApoE complex was found to decrease following loss or pharmacological disruption of TMEM97 or PGRMC1 (Riad et al., 2020). The results suggest that the S2R/TMEM97 complex is a binding site for Aβ42 on cell bodies and is critical for its cellular uptake of Aβ42 and ApoE. Furthermore, the complex may be a novel pharmacological target for inhibiting Aβ42 neuronal internalization, accumulation, and neurodegeneration and therefore suggests an approach to the treatment of AD. Astrocytes and glial cells also facilitate Aβ clearance through lysosomal degradation, although TMEM97 and PGRMC1 ligands bind more prominently and with higher affinity in neurons than in glia and the effect of S2R ligands on clearance from the brain through uptake by astrocytes and glial is unknown. It remains to be determined whether S2R inhibitors can be targeted exclusively toward the neuronal cell population *in vivo*, inhibiting neuron-specific Aβ uptake, accumulation, and neurodegeneration.

Substantial additional evidence for the role of S2R/TMEM97 in Aβ and AD comes from preclinical and clinical biomarker studies of the selective S2R allosteric antagonist CT1812. CT1812 is currently in clinical trials as a disease-modifying treatment for AD. Previously published literature indicates that Aβ oligomers bind to a multiprotein receptor complex composed of the proteins LilRB2, cellular prion protein, and NogoR (Kim et al., 2013; Smith et al., 2019) causing synaptotoxicity and cellular damage followed by cognitive decline in AD. Catalano, Izzo, and colleagues presented findings at the SfN meeting and at the S2R satellite symposium that are under peer review for journal publication at the time the present report was written (Catalano et al., 2019; Izzo et al., 2019). The findings suggest that the S2R receptor complex regulates the oligomer receptor complex on neurons. The binding of CT1812 to S2R likely modulates the conformation of S2R, which in turn allosterically alters the conformation of the oligomer binding pocket on oligomer receptors. Binding pocket destabilization leads to displacement of Aβ oligomers from neurons. Once displaced, Aβ oligomers are unable to rebind as long as threshold concentrations of CT1812 are present, as demonstrated in binding studies on neurons *in vitro*, in hippocampus of living AD transgenic mice *in vivo*, and in AD patient frozen postmortem neocortical brain tissue sections *ex vivo*. Consistent with previously published studies of closely related compounds (Izzo et al., 2014a,b), this effect on Aβ oligomers leads to synaptic restoration *in vitro* and improved performance in cognitive tasks in rodent AD models.

Results currently under peer review from a phase 1a/2b clinical trial of CT1812 in mild-to-moderate AD patients (*N* = 19; MMSE 18–26) were reported (ClinicalTrials.gov identifier: NCT02907567). Participants received one of three doses of CT1812 or placebo once daily for 28 d. Plasma and CSF were collected at baseline and following final dose administration, and protein, lipid, and metabolite values were measured using ELISA or tandem mass spectrometry. CSF concentrations of Aβ oligomers were found to be significantly increased in CT1812-treated patients compared with placebo-treated patients. This increase is consistent with preclinical studies demonstrating that CT1812 destabilization of the binding pocket on oligomer receptors leads to displacement of oligomers from neuronal surfaces and subsequent clearance into the CSF. Furthermore, CSF concentrations of fragments of the synaptic proteins neurogranin and synaptotagmin were significantly decreased in CT1812-treated versus placebo-treated patients, suggesting a reduction in synaptic damage with drug treatment.

Because both TMEM97 and PGRMC1 are known to impact lipid metabolism (Ahmed et al., 2012; Ebrahimi-Fakhari et al., 2016; Riad et al., 2018), plasma samples were analyzed for changes from baseline in a number of metabolites. Preliminary findings suggest that 11 individual metabolites that are known to be lowered in AD (Li et al., 2016; Toledo et al., 2017) were significantly altered from baseline in CT1812-treated versus placebo-treated patients and that 10 of these were elevated with drug treatment, consistent with a positive effect on disease course. In particular, lipid metabolites such as long chain polyunsaturated fatty acids as well as carnitines and acyl-carnitines decrease in AD (Toledo et al., 2017), whereas CT1812 treatment resulted in significant increases in these metabolites compared with placebo.

Together, these clinical data provide encouraging evidence of CT1812 target engagement in patients and are consistent with preclinical reports demonstrating that CT1812 and related S2R allosteric antagonists reduces synaptic damage and modifies disease biomarkers. The presented data are currently under peer review, and additional phase 2 six-month trials in this patient population are underway.

## The Role of the S2R Complex Protein PGRMC1 in Health and Pathology

Because S2R/TMEM97 forms a complex with PGRMC1, the value in pharmacologically targeting S2R includes the effects exerted through possible alteration of PGRMC1 activity, as well. The field of S2R research therefore includes understanding the role of PGRMC1 in health and pathology.

To illustrate the vast potential of PGRMC1 to impact heath and disease, Cahill and colleagues have worked to characterize the role of PGRMC1 in cell biology and cellular metabolic regulation. Evolutionary studies suggest that the PGRMC1 gene first originated in a bacterium and, following incorporation into eukaryotic cells, was involved in sterol production, hypothetically to modulate early mitochondrial oxygen response. This was perhaps related to a membrane trafficking motif and the ability to transfer sterols to mitochondria (as demonstrated through preliminary, unpublished findings). The eukaryotic PGRMC1-like family was originally defined by the presence of a variable number of residues inserted between two helices on the protein surface (Mifsud and Bateman, 2002). This region has high predicted propensity to form coiled-coil protein interactions and has recently been shown to share similarity with motifs in certain myosins (components of the actin cytoskeleton; Hehenberger et al., 2020). Furthermore, PGRMC1 can be found in complexes with components of the actin cytoskeleton (Salsano et al., 2020; Thejer et al., 2020a). Taken together, these results suggest that PGRMC1 modulation of the actin cytoskeleton could regulate actin-mediated mechanical forces required for vesicle trafficking. Modern PGRMC1 influences oxic/glycolytic and sterol metabolism, and membrane trafficking, which have all been associated with the σ receptors (Nicholson et al., 2016; Cahill and Medlock, 2017).

Evolutionary studies also revealed that the main PGRMC1 tyrosine phosphorylation sites (Y139 and Y180) appeared at the same time as the last eumetazoan common ancestor (LEUCA; common ancestor of cnidarians and bilaterally symmetrical animals). This was the first organism to possess a gastrulation organizer and postgastrulation differentiated cell types. Among these differentiated cells that first appeared in emetazoans were neurons (Hehenberger et al., 2020), suggesting an intimate and perhaps master-regulating role between PGRMC1 activity and neurogenesis, and perhaps adult neural state identity. Strikingly, one of the PGRMC1 phosphorylated tyrosines (Y139) is a coiled-coil heptad repeat residue in the center of the coiled-coil myosin-like motif (Hehenberger et al., 2020), suggesting immediately that its phosphorylation at gastrulation could (1) disrupt coiled-coil interactions, and (2) establish new phospho-tyrosine-dependent interactions with different proteins. This is striking because of the changes in actin-cytoskeleton associated with early gastrulation events (Patwari and Lee, 2008). In human cancer cells, mutation of PGRMC1 phosphorylation sites leads to changes in PI3K/Akt activity, glucose metabolism, epigenetic genomic CpG methylation, and mitochondrial structure and function, leading to attenuated cancer growth and alterations to signaling pathways associated with pattern establishment and cell differentiation. Results reported by Cahill at the symposium have since been published (Thejer et al., 2020a,b). Perturbations in glucose metabolism, epigenetics, and mitochondria are all symptoms of AD (Cenini and Voos, 2019; Ehrlich, 2019; Esposito and Sherr, 2019). Notably, gastrulation establishes the platform on which subsequent epigenetic determination of animal tissue-specific differentiated cell identity is based, and this may be related to changes in PGRMC1 function regulated by phosphorylation. We note that the same effects could direct synapse function.

It has long been known that a PGRMC1:deleted in colorectal carcinoma (DCC) interaction directs early embryonic axon guidance of central nerve cord neurons form nematodes to mammals (Cahill, 2007). We now know that PGRMC1 continues to function in adult synapses and is present in a protein complex that is the target of small molecule CT1812 which attenuates AD symptoms (reported here). We also know that DCC is critical in the mechanism of long-term potentiation, with both presynaptic and postsynaptic roles (Glasgow et al., 2018, 2020). Therefore, one simple hypothesis to explain the mechanism of action of CT1812 is that oligomeric Aβ engages the S2R/TMEM97/PGRMC1 complex in a state where PGRMC1 cannot contribute to DCC function, thereby preventing synaptic plasticity. This suggests an underappreciated role for PGRMC1 in AD pathogenesis. Indeed, the association of PGRMC1 with the complex that internalizes both Aβ and the genetic risk factor ApoE (Riad et al., 2020), and the association of Tau trafficking with that complex (Rodriguez-Vieitez and Nielsen, 2019; Yamazaki et al., 2019), potentially associates PGRMC1 biology with most of the main cell biological symptoms of AD (ApoE, Tau, Aβ, glycolysis, epigenetics, mitochondria, LTP), and with the mechanism of action of CT1812. We are unaware of another protein for which this claim can be made.

Furthermore, PGRMC1 plays an important role in mediating progesterone function and its associated neuroprotective effects. Nguyen et al., had previously reported that the miRNA let7i, which negatively regulates PGRMC1 expression, is upregulated following ischemic injury such as stroke (Nguyen et al., 2018). This results in a disruption of progesterone-induced BDNF release, reducing progesterone’s protective effect. Singh and colleagues are investigating whether inhibiting let7i will facilitate progesterone-mediated neuroprotection (Kim et al., 2019). To this end, they have used an H_2_O_2_-induced model of oxidative stress. At the SfN meeting and the S2R satellite symposium, Singh presented preliminary results from these studies that indicate an elevation in let7i expression in both the C6 astrocyte and SH-SY5Y neuronal cell lines, and a downregulation of PGRMC1, following H_2_O_2_-induced oxidative stress. Addition of a let7i inhibitor appears to reverse this negative effect and to restore the progesterone-mediated protection against oxidative stress in both cell types, as well as to enhance the protective efficacy of progesterone in an animal model of stroke (Nguyen et al., 2018). If further investigation confirms these findings, inhibitors of let7i may be an adjunctive therapy for neural injury and neurodegenerative disease. Given the relationship between PGRMC1 and S2R, the described influence of let7i may have relevance to the modulating neurobiology of S2R in health and disease.

## Development of S2R Ligands

Further development of S2R ligands will facilitate research into the receptor’s role in health and disease. Initial high-affinity ligands allowed pharmacological characterization of S2R activity and led to the understanding that it is present in many tumor types. Its ligands may also have a cytotoxic effect. In combination with the S2R role in AD, the therapeutic potential of ligands is significant and warrants their further development.

Abate and colleagues have employed a variety of approaches to generate these compounds and have produced fluorescent S2R ligands, nanoparticles that can be used as tools in S2R research, and multi-target agents intended to have cytotoxic effects (Abate et al., 2018, 2019). To accomplish these goals, the lead compounds were decorated, in the appropriate position, with alkyl linkers bridging the pharmacophore from either a fluorescent tag or a fluorescent nanoparticle as the quantum dot (Abate et al., 2011, 2014; Niso et al., 2015; Pati et al., 2018a). The resulting high-affinity fluorescent S2R agents are useful for non-radioactive binding assays and visualization of protein localization in living cells. In addition, multifunctional compounds were developed by connecting metal chelating moieties to S2R directing basic portions using alkyl linkers (Pati et al., 2015, 2018b).

Initial studies using these novel ligands revealed that S2R/TMEM97 binding is not needed for cytotoxic activity but increases the specificity of delivery and reduces toxic off-site activity. Together, these findings confirm that S2Rs are versatile targets worthy of continued investigation, and that S2R ligands should be developed through different strategies for use in diagnosis and treatment of diseases from tumors to neurologic disorders.

## Conclusions and Future Directions

S2R is involved in a number of biological processes relevant to both health and disease. Understanding of S2R pharmacology and biology has improved to a noteworthy degree in recent years, but a number of outstanding questions remain. Further research and compound development are needed to ensure the full potential of this receptor as a therapeutic target in cancer and neurodegeneration is realized. The annual International Symposium on Sigma-2 Receptors at the SfN conference ensures that researchers focused on this versatile receptor and its associated proteins are sharing progress and collaboratively moving the field forward.

## Synthesis

Reviewing Editor: Karl Herrup, University of Pittsburgh School of Medicine Decisions are customarily a result of the Reviewing Editor and the peer reviewers coming together and discussing their recommendations until a consensus is reached. When revisions are invited, a fact-based synthesis statement explaining their decision and outlining what is needed to prepare a revision will be listed below. The following reviewer(s) agreed to reveal their identity: NONE.

The authors are to be complimented for the thoughtful and robust ways in which they addressed the concerns raised in the original review. No further changes are required.
